# Christmas at the Hospitals

**Published:** 1891-12-19

**Authors:** 


### Christmas at the Hospitals.

### THE FARE AND ENTERTAINMENTS.

Christmas affords hospital Governors and others interested in their work and welfare an exceptional opportunity of seeing what is being done in a practical way for the sick and suffering. The following list of the entertainments and fare which will be provided for the patients in the Metropolitan hospitals has been supplied on authority, and will enable anyone interested to pay a visit to one or more of the institutions with the result, it is to be hoped, that they may be induced to give liberally of their abundance towards the estimated deficiency of £150,000 which the authorities will have to face during the current year, owing to the exceptional depression which has characterised 1891. We are assured that visitors will be welcomed, and shall be very glad to receive an account of their experiences and the impressions gained from their visit.

**Alexandra Hospital,** Queen's Square, W.C.

There are no Christmas festivities, but to make up for this a treat to the children is arranged for in January.

**Belgrave Hospital for Children,** Gloucester Street, S.W.

Early in January the Christmas tree will be held, all the presents being placed in the "Princess Beatrice" Ward. All the children who are well enough will be brought here to see it, and many of the little out-patients will be invited to the treat. The presents, consisting of toys of all kinds, crackers, and woollen garments, will then be distributed to all the children. After this the out-patients will be given a nice tea, and all will adjourn to a magic lantern entertainment, those in-patients who are well enough being carried down to see it also. H.R.H. Princess Beatrice is the Lady Patroness, and never forgets to send some toys for the little ones each Christmas.

**East London Hospital for Children,** Shadwell, E.

Christmas trees will be provided for the patients of this hospital in the various wards, and it is hoped that a good supply of toys will be received with Christmas cheer to brighten Christmas in the hospital. Those who are well enough will enjoy a Punch and Judy show in the board-room, while, through the kindness of friends, the nurses and staff will be provided with a concert and special supper afterwards.

**Evelina Hospital for Sick Children,** Southwark Bridge Road, S.E.

In this institution the children's fête does not come off till January 14th.

**King's College Hospital.**

The wards of the hospital will be tastefully decorated. Every patient will receive Christmas cards, &c., and presents of warm clothing will be given. The children will have toys in abundance. Christmas dinners will be served to all who may be able to eat Christmas fare, but the chief feature of the festivities is the Christmas tea at four o'clock, when the wards will be brilliantly illuminated. After tea the men are allowed to smoke and to have a moderate allowance of whiskey, the Resident Medical Officers kindly providing the tobacco and whiskey. Patients frequently say that their Christmas in hospital has been one of their happiest. The Warden recollects one patient telling him he had never spent such a happy Christmas Day before. "You see, Sir," said he, "yesterday was the fust Christmas Day as I have been sober for years."

**London Hospital,** Whitechapel, E.

Children's Christmas trees will be held on the January 5th and, "Mr. Punch's" enthralling adventures will be performed. Tea will be provided for visitors in the Nursing Home from 3.30 to 5.30.

**London Temperance Hospital,** Hampstead Road, N.W.

According to rule, those of the patients who are able to eat such fare, are given a Christmas dinner of turkey, plum-pudding, and dessert. Each patient will be allowed to invite a friend to tea, and for the evening various amusements are provided. Dr. Collins has kindly secured the services of a well-known conjuror, and a Christmas tree will furnish presents of a useful character to all patients and members of the household. Refreshments will be handed round at intervals. An entertainment for patients and staff will take place on December 28th, when the Rev. Canon Fleming has promised to give some readings, and other friends have undertaken to contribute songs and instrumental music.

**Metropolitan Hospital,** Kingsland Road, E.

Christmas will commence at an early hour with carols and Christmas hymns, and everybody in the building, down to the scrubbers, will receive a present. The day and night nurses will then sit down to breakfast together, the charge nurses looking after the wards meanwhile. Later on, a gaily-dressed Christmas tree in the children's ward will absorb the attention of the eager little people, and the evening will bring with it songs and merriment. Early in the New Year there will be a concert, and, perhaps, theatricals. The exact form which the entertainment will take this year is not, however, definitely settled.

**The Middlesex Hospital, W.**

On Christmas day tea-parties will be given in all the wards to the patients, their friends, and the nursing staff. There will be a gigantic Christmas tree in the Board-room, and at 3 p.m. a distribution of the Christmas gifts will take place. In the nurses' home there will also be a friendly gathering of nurses, Sisters, and lady, and other probationers, and their friends. On New Year's day a Christmas tree party will be given in the children's ward.

**National Hospital for Paralysed and Epileptic,** Queen's Square. W.C.

There is an entertainment provided for the patients every fortnight, from November to Easter, by the staff, medical and otherwise, of the hospital. At Christmas there is a special entertainment and a Christmas tree, and in addition small entertainments are given by the staff nurses of each ward to their patients—each ward trying to have the most varied programme and be thought the best of the season.

**Paddington Green Children's Hospital.**

It is purposed to give an entertainment in the out-patient room of this hospital on Thursday, December 31st. There will be present about 150 children who have been in-patients during the past year and who have since returned to their homes. The entertainment will consist of a tea, a magic lantern entertainment, and a present of toys. All the members of the staff will be present.

**Royal London Ophthalmic Hospital;** Moorfields, E.C.

On Christmas Day the patients will be provided with roast beef and plum pudding, afterwards fruit of all kinds sent by kind donors. The men have gifts of tobacco, and an extra allowance of beer or tea. On the 30th it is proposed to have a dance (Cinderella) for the Sisters and nurses. On the 22nd of January a concert will be given by the Matron and house surgeons to the in-patients, Sisters, and nurses.

**St. George's Hospital,** Hyde Park Corner, S.W.

Owing to the large majority of the cases being very ill and unable to be up, the Christmas entertainments to the patients are compelled to be held in the wards. On Christmas Day a dinner of roast beef and plum pudding is provided. On the following evening the ladies who visit the wards will give the patients a tea, when they will be supplied with cakes, oranges, &c., and when the ladies will do their best to amuse them, due consideration being, of course, given to those patients whom it is necessary shall be as little disturbed as possible. The entertainment to the nursing staff will take place early in January.

**St. Mary's Hospital,** London, W.

This institution has two Christmas trees for the children on different nights, one in the surgical and one in the medical ward. An extra treat, a Punch and Judy show, will be provided, and the gifts on the Christmas tree will be given away by some friend dressed up as Father Christmas. In the beginning of the New Year an entertainment for the nurses and their friends, and one for the patients, will also be given. The wards will be decorated on Christmas Eve with the help of friends.

**St. Thomas's Hospital,** Albert Embankment, S.E.

On Christmas Day the usual service will be held in the chape at 10.30 a.m.; the patients' dinner of roast beef and plum pudding, and dessert at noon. The men will be allowed to smoke during the afternoon. The Nightingale Nurses will visit each ward during the evening, and sing Christmas Carols, &c. The Sisters, Nurses, Probationers, &c., will also be provided with Christmas Cheer. About the middle of January next a theatrical performance will be given in one of the theatres, and a few days later a concert in the Governors' Hall for the Sisters and nurses will be held.

**Samaritan Free Hospital for Women and Children,** Marylebone Road, N.W.

Here the Christmas entertainment does not come off till the 31st inst.

**Westminster Hospital,** Broad Sanctuary, S.W.

The usual Christmas fare will be provided, and in the afternoon and evening friends will attend at the hospital, and do their best to make the day pass happily and pleasantly. The men will be provided with tobacco, the women will receive gifts of clothing, and the children toys.

We invited information from all the hospitals, but have only been able to give that which came to hand in time for this week's issue.

